# Assessment of the efficacy of SARS-CoV-2 vaccines in non-human primate studies: a systematic review

**DOI:** 10.12688/openreseurope.14375.1

**Published:** 2022-01-07

**Authors:** Michel Jacques Counotte, Mariana Avelino de Souza Santos, Koert J Stittelaar, Wim H M van der Poel, Jose L Gonzales

**Affiliations:** 1 Wageningen Bioveterinary Research, Wageningen University and Research, Lelystad, The Netherlands

**Keywords:** SARS-CoV-2, Animal models, Preclinical data, Vaccine efficacy

## Abstract

**Background: **The outbreak of Severe acute respiratory syndrome coronavirus 2 (SARS-CoV-2) triggered the rapid and successful development of vaccines to help mitigate the effect of COVID-19 and circulation of the virus. Vaccine efficacy is often defined as capacity of vaccines to prevent (severe) disease. However, the efficacy to prevent transmission or infectiousness is equally important at a population level. This is not routinely assessed in clinical trials. Preclinical vaccine trials provide a wealth of information about the presence and persistence of viruses in different anatomical sites.

**Methods: **We systematically reviewed all available preclinical SARS-CoV-2 candidate vaccine studies where non-human primates were challenged after vaccination (PROSPERO registration: CRD42021231199). We extracted the underlying data, and recalculated the reduction in viral shedding. We summarized the efficacy of  vaccines to reduce viral RNA shedding after challenge by standardizing and stratifying the results by different anatomical sites and diagnostic methods. We considered shedding of viral RNA as a proxy measure for infectiousness.

**Results: **We found a marked heterogeneity between the studies in the experimental design and the assessment of the outcomes. The best performing vaccine candidate per study caused only low (6 out of 12 studies), or moderate (5 out of 12) reduction of viral genomic RNA, and low (5 out of 11 studies) or moderate (3 out of 11 studies) reduction of subgenomic RNA in the upper respiratory tract, as assessed with nasal samples.

**Conclusions: **Since most of the tested vaccines only triggered a low or moderate reduction of viral RNA in the upper respiratory tract, we need to consider that most SARS-CoV-2 vaccines that protect against disease might not fully protect against infectiousness and vaccinated individuals might still contribute to SARS-CoV-2 transmission. Careful assessment of secondary attack rates from vaccinated individuals is warranted. Standardization in design and reporting of preclinical trials is necessary.

## Research in context

### Evidence before this study

Vaccine efficacy is often defined as the proportion of a vaccinated individuals to not fall ill after infection. However, its ability to curb transmission is equally important, especially during early vaccine roll-out when many people are still susceptible to infection. For most SARS-CoV-2 vaccines that are on the market, the vaccine efficacy to prevent disease is good, however, much is still unclear about their ability to prevent infectiousness. We assessed the protocols of the clinical trials (clinicaltrials.gov and covid19.trackvaccines.org) and performed a PubMed and EMBASE search on ‘vaccine efficacy’ and ‘SARS-CoV-2’. The clinical trials in which these vaccines were tested, did not assess infectiousness or viral shedding over time, and were underpowered to produce, for example, estimates of secondary attack rates. Similarly, to date, no large studies have compared the secondary attack rates between vaccinated and unvaccinated individuals. It remains unknown and debated how effective these vaccines are to lower infectiousness and prevent SARS-CoV-2 transmission.

### Added value of this study

To our knowledge, this is the first systematic review and re-analysis of the underlying data of preclinical challenge studies that assessed the efficacy of SARS-CoV-2 vaccine candidates. Animal models allow rigorous and systematic assessment of viral shedding over time and provide evidence on vaccine efficacy that is often overlooked.

### Implications of all the available evidence

Many of the candidate vaccines only triggered a low reduction of shedding of viral RNA in the upper respiratory tract. This calls for caution, since these SARS-CoV-2 vaccines might not fully protect against infectiousness. Standardization of trial design and reporting will help contribute to the comparability and interpretation of preclinical trials.

## Introduction

The pandemic of severe acute respiratory syndrome coronavirus 2 (SARS-CoV-2) that causes coronavirus disease 2019 (COVID-19) prompted an outbreak response without precedent. To mitigate the devastating effect of the disease, vaccine development has been rapid and successful. In under a year after the World Health Organization declared the outbreak a public health emergency of international concern, the first SARS-CoV-2 vaccine (Pfizer-BioNTech) had received emergency use authorization by the United States Food and Drug Administration. In China and Russia emergency access to the first vaccines was already granted mid-2020; the ‘ CoronaVac’ vaccine received approval for emergency use in July 2020 ^1^, and the Russian Sputnik V vaccine received emergency authorization in early August 2020 ^2^. As of March 26, 2021, there were 92 vaccine candidates in trials, of which 13 had received approval in one or more countries.

An important metric to quantify the performance of a vaccine is the ‘vaccine efficacy’, which, in a clinical setting, is often defined as capacity of a vaccine to prevent (severe) disease ^3^. However, vaccine efficacy is not only reflected by the prevention of symptomatic disease after infection, but also the effect on the susceptibility and infectiousness ^4, 5^, e.g., the ability of a virus to establish infection in a host and the ability of a host to infect others. As Lipsitch and Dean (2020) comprehensively explain, there is a necessity to look beyond the effect of a vaccine on an individual, and also examine the efficacy in the context of preventing transmission ^6^. Where frontline health workers are among the first to be vaccinated, Lipsitch and Dean note that “A worst-case scenario is a vaccine that reduces disease while permitting viral shedding; this could fail to reduce transmission or conceivably even increase transmission if it suppressed symptoms.”. Even though vaccination might reduce the severity of disease, the vaccination effect on the risk of transmission also needs to be considered. Individuals, despite having an asymptomatic SARS-CoV-2 infection, can still transmit virus ^7^. Similarly, this could mean that infected vaccinated people, despite not showing any symptoms or even being aware of their infection status, pose a risk to others. Ongoing transmission under this scenario could lead to emergence of new virus variants.

The typical development cycle of any vaccine includes an assessment of safety, immunogenicity and efficacy in preclinical animal models ^8^. Successful candidates are then trialled in humans during different phases. Preclinical studies already provide a wealth of information regarding the vaccine efficacy and transmission dynamics. Several animal models have been established in both small animals like mice, hamsters, and ferrets, and larger animals such as non-human primates (NHP) ^9^.

Early in the pandemic, NHP models were established. Experimental infection of Cynomolgus macaques ( *Macaca fascicularus*), with SARS-CoV-2, caused COVID-19-like disease and viral shedding in the nose and throat ^10^. Similarly, a rhesus macaque ( *Macaca mulatta*) model was established, that mimicked human infection characteristics, however, with less marked clinical signs ^11^. Both models allowed assessment of viral shedding kinetics in the upper and lower respiratory tract. Especially for vaccine development, challenge studies in these animal models provide insight in the efficacy of the vaccines to prevent virus replication and illness, but most importantly on their effect on infectiousness by measuring viral shedding over time; a measurement that is much harder to obtain in human field trials.

Here, we present a systematic assessment and comparison of the preclinical SARS-CoV-2 vaccine evaluation studies in NHPs to provide insight in the vaccine efficacy of the candidate vaccines.

## Methods

### Research question

**Figure 1.**
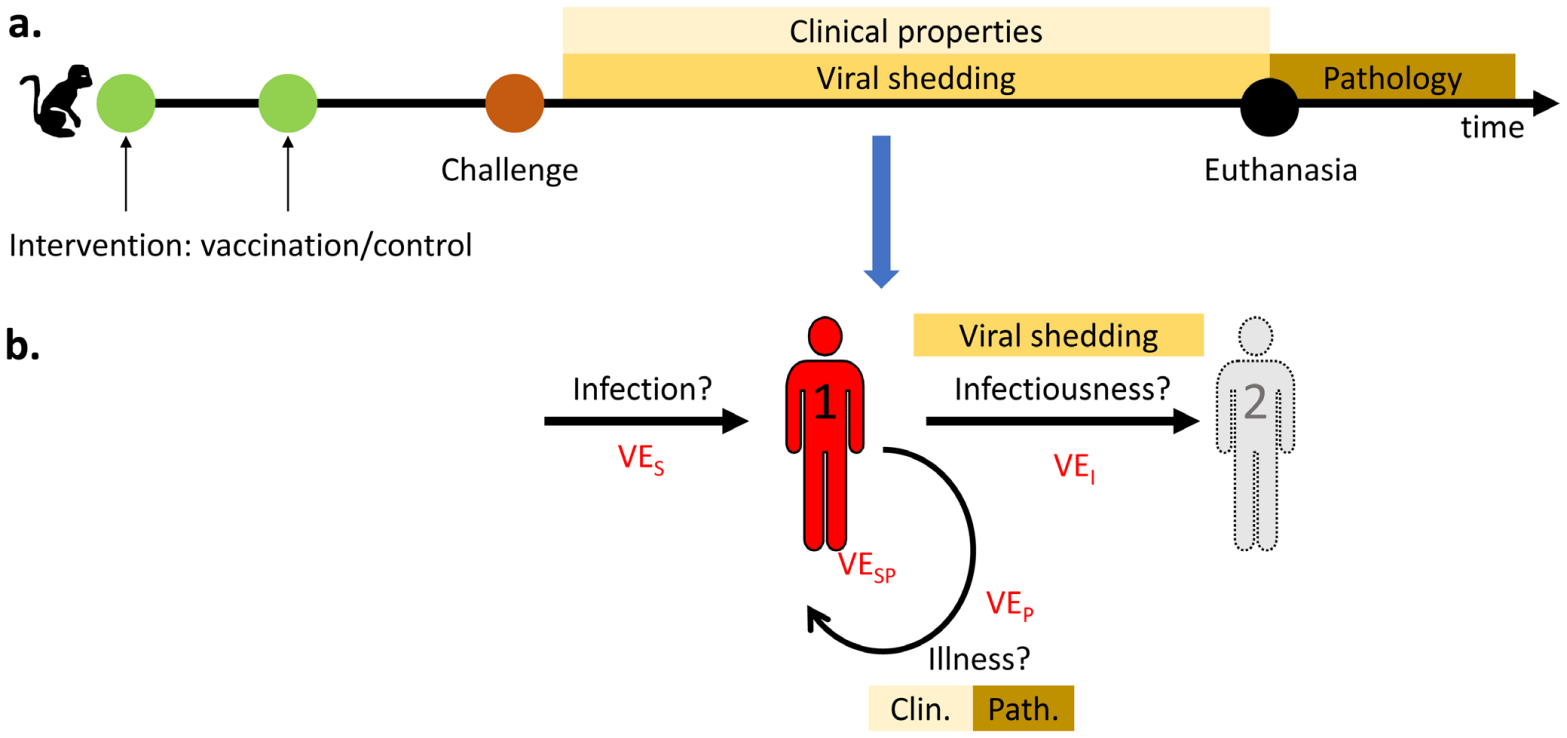
The relationship between the challenge experiments’ outcomes ( **A**) and the vaccine efficacy ( **B**). ( **A**) The timeline of a typical non-human primate vaccination-challenge experiment. After challenge, viral shedding and clinical parameters are collected until time of euthanasia, after which pathological examination is performed. ( **B**) Vaccine efficacy (VE) can help: (i) Protect an individual (1) against infection (VE _S_), (ii) Prevent infectiousness (VE _I_) and prevent transmission to a second individual (2) for which viral shedding can be a proxy measure, (iii) Prevent illness (VE _P_) for which clinical or pathological findings can serve as indicator.

We defined the elements of the research question by PICO (Population, Intervention, Control, Outcome) question ^12^: The population of interest was defined as ‘any non-human primates’. The intervention of interest as ‘vaccination against SARS-CoV-2, followed by a challenge experiment’; experiments needed to compare against a control group. The outcome of interest was ‘vaccine efficacy’. We use the reduction in shedding of viral RNA as proxy for the vaccine efficacy to prevent infectiousness ( Figure 1). The reduction in clinical and pathological findings is interpreted as the vaccine efficacy to prevent disease.

### Search strategy and selection criteria

We searched the electronic databases PubMed, EMBASE, BioRxiv, and MedRxiv as indexed by the ‘COVID Open Access Project’ ^13^. We filtered the dataset of COVID-19 related research based on keywords in the title and or abstract: “(animal OR mouse OR mice OR hamster OR macaque OR primate OR monkey OR preclinical OR pre-clinical) AND (vaccine OR immunization OR vaccination)”. The full review protocol is available on PROSPERO (registration: CRD42021231199).

We imported the deduplicated search results into the online screening tool ‘Rayyan’ ^14^. Additionally to the search-strategy, we checked the reference lists of the included studies and review articles on the same topic for relevant publications. We included studies published before January 20, 2021.

We included studies that assessed COVID-19 vaccine candidates in preclinical NHP models, and that conducted a challenge study in both preprint and peer-reviewed literature. We included animal models in any of the following species: Rhesus macaques ( *Macaca mulatta*), cynomolgus macaques ( *Macaca fascicularis*), African green monkeys ( *Chlorocebus aethiops*), and other non-human primates. We included animal models regardless of the sex any age of the animals. We only included studies that compared a control group with the vaccinated group or groups. Studies that did not provide extractable data on the presence of viral RNA after challenge were excluded.

Two reviewers screened titles and abstracts of the retrieved papers independently. If retained, we assessed the full text of the study. One reviewer extracted data into piloted extraction forms in Excel (version 16.0.12527). A second reviewer reviewed exclusion decisions and data entry. Disagreement was resolved by discussion between the reviewers.

### Data extraction

We extracted the full citation information, and relevant information on the methods used in the different studies (the full data is available in Supplementary Material Text 1). We extracted the results either from the text, figures or the supplementary data files. For studies that did not provide the full data, we used WebPlotDigitizer to extract the data from the figures. We collected the type of RNA that was assessed (subgenomic or genomic), from which anatomical sites the samples came, and the raw values of number of viral RNA copies at all provided time-points. For the reported results of vaccination on reduction in pathological and clinical changes, we summarized the direction of the effect (increased, null, or reduced) and whether the reporting was qualitative or quantitative.

To allow the comparison between studies, we standardized the nomenclature of groups. Groups that were challenged, but that did not receive a vaccine are considered ‘control’ groups, we provide the corresponding name of the intervention groups in the Supplementary Material Table 1.

We also standardized the anatomical sites where samples were taken. We grouped nasal swabs, nasal washes, and nasal brushes as “nasal samples (NS)”, tracheal swab and tracheal brushes as “tracheal samples (TS)”. Anal and rectal swabs were grouped as “anal samples”. We assumed that the nasal and tracheal samples provided information on the upper respiratory tract; the BAL provided insight in the presence of virus in the lower respiratory tract. Anal samples provided proof of systemic infection. To standardize the challenge dose in plaque forming units (PFU), we assumed that the Median Tissue Culture Infectious Dose (TCID50) was approximated by halve of the PFU value ^15^.

### Synthesis of the evidence

We provided a narrative synthesis of the methodology of the different challenge experiments. For the outcome ‘vaccine efficacy to reduce infectiousness’, we used the reduction of shedding of RNA as measured by RT-PCR in the different anatomical sites as a proxy measure. We assessed the relative reduction in viral RNA shedding by calculating the difference in AUC between the vaccinated group and the control group. We defined the relative difference as the AUC of the vaccinated group minus the AUC of the control group, divided by the AUC of the control group. For visualization purposes we grouped the difference in AUC using ‘Jenks natural breaks’ into three groups: low (<18%), moderate (18-44%) and high (>44%) reduction of viral RNA excretion ^16^. We explored the relationship between relative reduction in AUC and the challenge dose visually. All analyses were performed using R (version 4.0.3). We assessed the efficacy in the prevention of disease, by summarizing the reported results on clinical outcomes and pathological changes. We tabulated the description of the outcome, and whether the outcome was quantitatively or qualitatively described. We report this review following the Preferred Reporting Items for Systematic review and Meta-Analysis (PRISMA) ^17^.

## Results

### Description of the included studies

We performed a systematic review of the literature indexed in PubMed, EMBASE, BioRxiv and MedRix until January 20, 2021. We identified and screened 742 studies of which we included 147 based on title and abstract (Extended data: PRISMA: flowchart & checklist). After the screening of the full text, we retained 21 studies ^18– 38^.

In the retained studies, that describe preclinical challenge experiment in non-human primates, a total of 16 studies used rhesus macaques ^18, 20– 23, 25– 28, 31, 33– 38^ five used cynomolgus macaques ^19, 24, 29, 30, 32^ The age of the animals ranged between 2–22 years; the majority of animals were young adults (Supplementary Material Figure 1). On average two vaccinations (range: 1–7) per study were tested. These were either different formulations or different doses (Supplementary Material Table 1). The animals were challenged after vaccination at a median duration of 28 days (range: 8–91 days) after administration of the last vaccine. Challenges were administered at different doses, in different volumes, via different routes: In the majority of studies, the animals (13 out of 21) were challenged both intranasally and intratracheally. In five studies, animals were challenged only either intranasally or intratracheally. In Table 1 we provide an overview of the experimental methods of the included studies. Additional properties of the vaccines are provided in Supplementary Material Table 2.

**Table 1.** Methodological properties of the included studies. The manufacturer, name, type, dosage, timing, and route of the vaccines used. The timing, route and SARS-CoV-2 strain of the challenge used. *Timing of challenge is defined as the time between the last vaccination and challenge. Abbreviations: IM: intramuscular, IT: intratracheal, IN: intranasal, SC: subcutaneous, ID: intradermal, PO: *per os*/ oral, IV: intravenous, PFU: plaque-forming units, TCID50: 50% tissue culture infective dose, VP: viral particles, wk: week, d: day.

Author [ref.]	Manufacturer	Type of vaccine	Species (macaque)	Age (year)	Challenge dose	Timing of challenge *	Challenge route	Challenge strain
Bewley ^18^	unknown	Inactivated	rhesus	2–4	5x10 ^6^ PFU	d14	IT, IN	SARS-CoV-2 Victoria/01/2020
Brouwer ^19^	unknown	Subunit (nanoparticle- based)	cynomolgus	5–6	10 ^6^ PFU	d14	IT, IN	hCoV-19/France/ lDF0372/2020
Corbett ^20^	Moderna	mRNA	rhesus	3–6	7.6x10 ^5^ PFU	d28	IT, IN	SARS-CoV-2 USA-WA1/2020
Feng ^21^	CanSino	Vector: Adeno	rhesus	6–14	2x10 ^4^ or 400 TCID50	d30, d56	IT	2019-nCoV-WIV04
Gabitzsch ^22^	ImmunityBio	Vector: Adeno	rhesus	>2.5	10 ^6^ TCID50	d28	IT, IN	not reported
Gao ^23^	Sinovac	Inactivated	rhesus	3–4	10 ^6^ TCID50	d8	IT	SARS-CoV-2 CN1
Guebre-Xabier ^24^	Novavax	Subunit	cynomolgus	>3	1.1x10 ^4^ PFU	d14	IT, IN	2019-nCoV/USA-WA1/2020
Liang ^25^	Clover Biopharmaceuticals	Subunit	rhesus	3–6	2.6x10 ^6^ TCID50	d14	IT, IN	107 (Guangdong Provincial CDC, Guangdong, China)
Mercado ^26^	Janssen Vaccines & Prevention	Vector: Adeno	rhesus	6–12	1.0x10 ^5^ TCID50	d42	IT, IN	USA-WA1/2020
Patel ^27^	Inovio	DNA	rhesus	unknown	1.1x10 ^4^ PFU (1.2x10 ^8^ vp)	d91	IT, IN	USA-WA1/2020
Rauch ^28^	CureVac	mRNA	rhesus	3–6	5x10 ^6^ PFU	d28	IT, IN	VERO/hSLAM cell passage 3 (Victoria/1/2020)
Sanchez-Felipe ^29^	KU Leuven	Vector: Yellow-fever	cynomolgus	unknown	1.5x10 ^4^ TCID50	d14	IT, IN	not reported
Seo ^30^	Genexine/VaxiGen	DNA	cynomolgus	5–6	2.6x10 ^7^ TCID50	d35	IT, IN, PO, IV, conjunctival	SARS-CoV-2 virus, obtained from the National Culture Collection for Pathogens (accession number 43326)
Solforosi ^31^	Janssen Vaccines & Prevention	Vector: Adeno	rhesus	13–22	1x10 ^5^ TCID50	d35, d91	IT, IN	G614 SARS-CoV-2
Sun ^32^	unknown	Subunit	cynomolgus	>3	10 ^6^ TCID50/mL (3.2ml)	d28	IT, IN, ocular	BetaCoV(BJ01)
van Doremalen ^33^	Astrazeneca/Oxford	Vector: Adeno	rhesus	2–4	4x10 ^5^ TCID50/ml (6.5ml total)	d28	IT, IN, PO, ocular	nCoV-WA1-2020
van Doremalen2 ^34^	Astrazeneca/Oxford	Vector: Adeno	rhesus	4–11	10 ^6^ TCID50	d28	IT, IN	SARS-CoV-2/human/USA/RML-7/2020
Vogel ^35^	Pfizer/BioNTech	mRNA	rhesus	2–4	1.05x10 ^6^ PFU	d55	IT, IN	USA-WA1/2020
Wang ^36^	Sinopharm	Inactivated	rhesus	2–4	10 ^6^ TCID50	d10	IT	not reported
Yang ^37^	unknown	Subunit	rhesus	5–9	0.5 ml, 10 ^6^ PFU/ml (5x10 ^5^ PFU)	d21	IN	not reported
Yu ^38^	Harvard/Janssen Vaccines & Prevention	DNA	rhesus	6–12	1.2x10 ^8^ vp (1.1x10 ^4^ PFU)	d21	IT, IN	not reported

### Reduction in excretion of viral RNA

For the quantification of viral RNA in the different anatomical sites after challenge, the data of individual animals was provided for seven studies ^20– 22, 26, 29, 33, 36^. For the remaining 14 studies we extracted the aggregated results from the text or the figures ^18, 19, 23– 25, 27, 28, 30– 32, 34, 35, 37, 38^. All studies reported measurements of the presence of viral RNA (genomic or subgenomic) at multiple timepoints. Animals were followed-up after challenge for a median period of seven days (range: 4–14 days). In most studies (17 out of 21) the presence of viral RNA in the upper respiratory airways was detected either by using nasal samples ^18– 22, 24– 28, 30– 35, 38^ or by tracheal samples (10 out of 21) ^18, 19, 23, 25, 29– 32, 36, 37^, To detect viral RNA in the lower airways, bronchoalveolar lavage (BAL) was used in 10 out of 21 studies ^20, 22, 24, 26, 27, 31, 33– 35, 38^. Seven studies assessed the viral excretion using anal swabs ^19, 23, 25, 32, 35– 37^.

**Figure 2.**
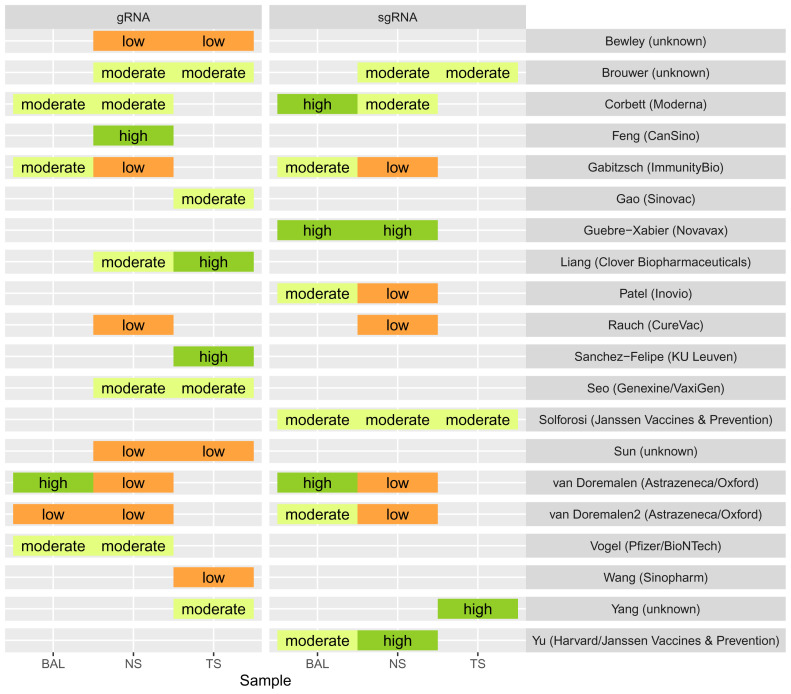
Reduction in viral genomic and subgenomic RNA after vaccination in different anatomical sites, expressed as relative difference in area under the curve (AUC). The best performing vaccines are shown by study; control groups serve as reference group for the comparison and are thus omitted from the figure. Labels show the first author of the study and the vaccine manufacturer in brackets. For visualization purposes we grouped the difference in AUC using ‘Jenks natural breaks’ into three groups: low (<18%), moderate (18–44%) and high (>44%) reduction of viral RNA excretion. Abbreviations: gRNA, genomic ribonucleic acid (RNA); sgRNA, subgenomic RNA; BAL, Bronchioalveolar lavage; NS, nasal or nasopharyngeal swab; TS, tracheal swab or tracheal brush.

**Figure 3.**
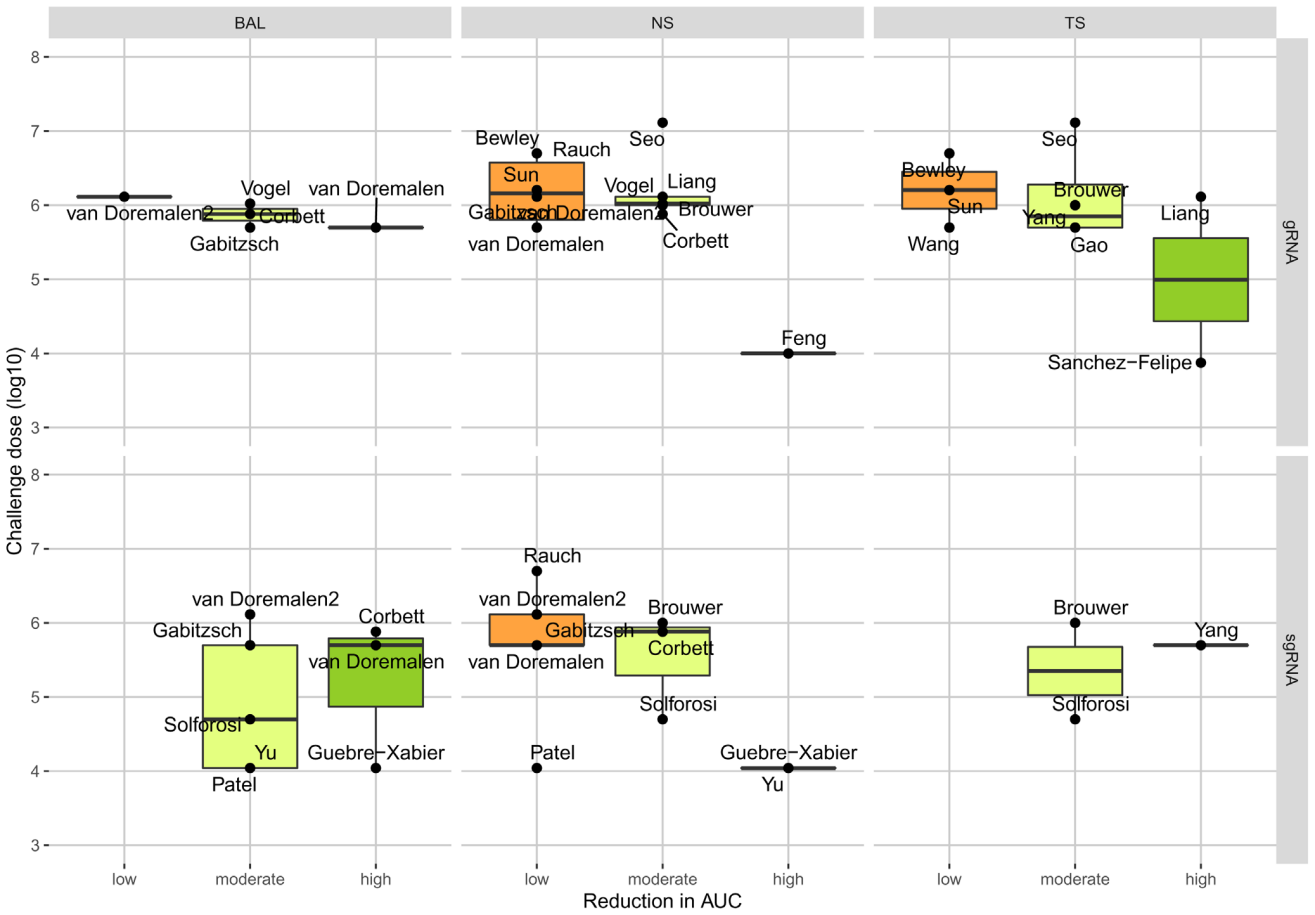
The relationship between challenge dose and reduction in area under the curve (AUC) between the best-performing vaccine per study in different anatomical sites. Abbreviations: gRNA, genomic ribonucleic acid (RNA); sgRNA, subgenomic RNA; BAL, Bronchioalveolar lavage; NS, nasal or nasopharyngeal swab; TS, tracheal swab or tracheal brush.

In the upper respiratory tract, as assessed with nasal samples, the best performing vaccine candidate per study caused only a low (6 out of 12 studies), or moderate (5 out of 12) reduction of viral genomic RNA, and low (5 out of 11 studies) or moderate (3 out of 11 studies) reduction of subgenomic RNA. Figure 2 provides an overview of the relative reduction of excretion of viral RNA in the different samples for the best performing vaccine candidates by study. A full overview of all tested vaccines is provided in Supplementary Material Figure 2. We observed a negative trend between the challenge dose and the reduction of viral genomic RNA ( Figure 3). We did not see a trend for the age of the animals used and the time between last vaccination and challenge (Supplementary Material Figure 3 & 4).

### Reduction in clinical and pathological outcomes

**Figure 4.**
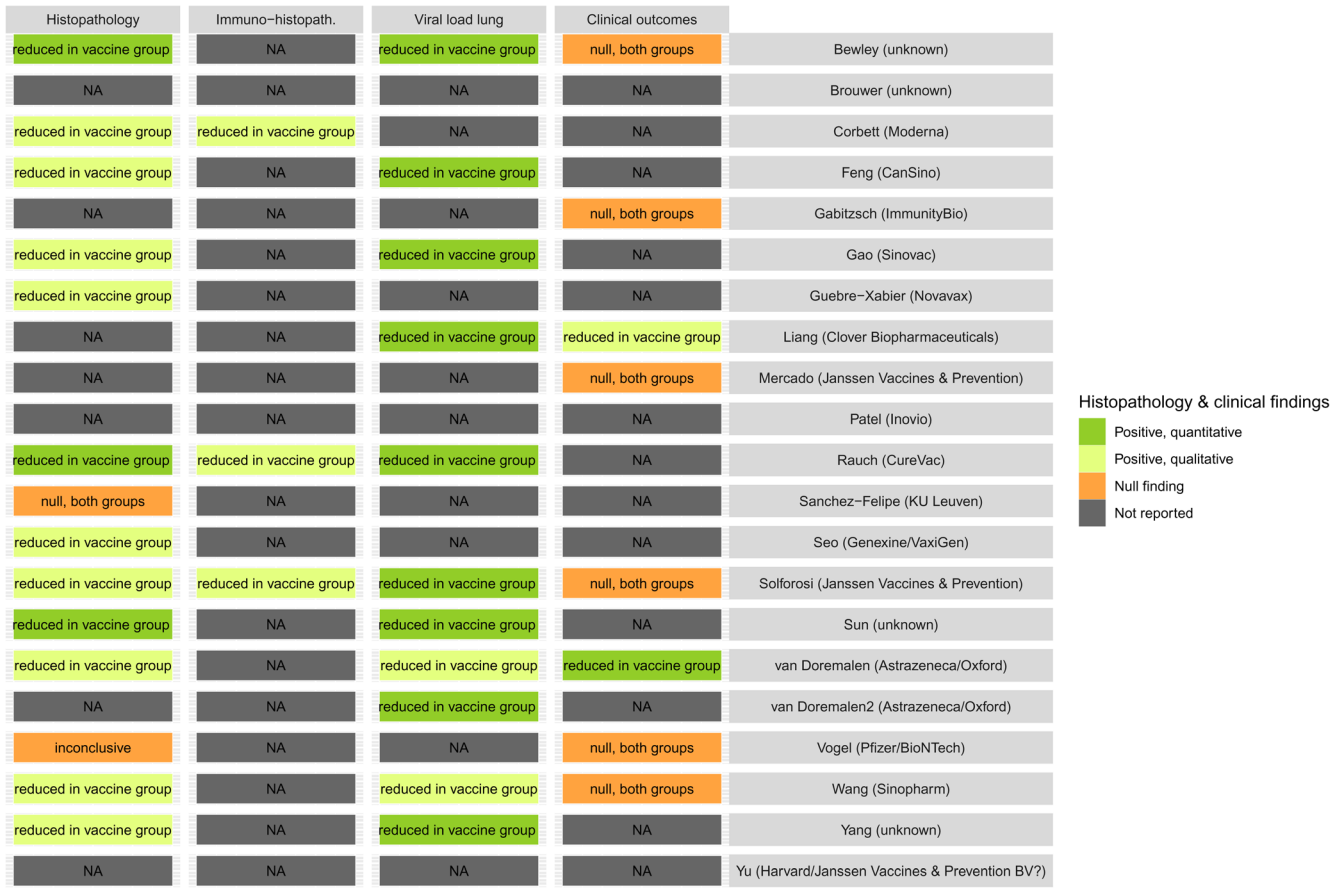
Summary of pathological and clinical results from the included preclinical vaccine studies. Abbreviations: NA, not available.

Reports on clinical and pathological outcomes were used as indicators of the potential efficacy of the vaccine candidates to reduce or prevent disease. Out of the 21 included studies, 14 reported histopathological findings ^18, 20, 21, 23, 24, 28– 33, 35– 37^ and eight reported on the clinical status of the animals ^18, 22, 25, 26, 31, 33, 35, 36^. The reporting was often qualitative ( Figure 4), and clinical symptoms were seldom reported, which may indicate the difficulty of monitoring clinical signs in these animal models. Of the 14 studies reporting pathological findings, 12 indicated a reduction in severity (histopathology lesions) in the vaccinated group. Whilst clinical status was reported in eight studies only two reported a reduction in clinical manifestation. All studies that reported a reduction in viral load in the lung also reported (qualitatively or quantitatively) a reduction in histopathological lesions. The data did not allow a quantitative comparison of the results.

## Discussion

Here, we provided a systematic and standardized summary of the results of preclinical SARS-CoV-2 vaccine trials in NHPs, where animals were challenged after vaccination. There was a strong heterogeneity in the methodology and reporting between studies, hampering comparability of the candidate vaccines. However, we were able to summarize the efficacy of the vaccines to reduce viral RNA shedding after challenge by standardizing and stratifying the results by different anatomical sites and diagnostic method. We found that vaccination with many of the candidate vaccines only seem to result in a low or moderate reduction of the shedding of viral RNA in the upper respiratory tract as detected in nasal swabs. The effect on the clinical parameters and histological findings were often only reported qualitatively.

A strength of this review is that we standardized the results of preclinical NHP vaccine models where possible, and visualized the remaining heterogeneity. Compared to other published reviews on the same topic, we are the first to provide a systematic quantitative analysis of the data that facilitates the interpretation and future trial design, where in other reviews, the results of preclinical vaccine models have only been summarized narratively ^8^.

Our review also has limitations. First, we chose to assess the vaccine efficacy only in NHP models. Despite being closest to humans on many aspects, the NHP models do not mimic the COVID-19 clinical disease in humans ^35^. However, the profile of viral shedding, which is the most relevant proxy measure of infectiousness, seems similar ^9, 10, 39^. Second, the way we calculated the area under the curve (AUC) was hampered by the reporting quality of the studies. The absence of viral RNA cannot be measured other than that the value falls below a certain limit of detection. This threshold varies between techniques and the way the data is visually presented differs between studies. For example, studies can plot points at the limit of detection, or some arbitrary value to ‘aid’ visualization: Yang *et al.* impute artificial zero’s ^37^ and Vogel *et al.* chose to plot values below the limit of detection at halve the value of that limit ^35^. No access to the full data, hence relying on digitizing images, creates an artificial ‘baseline’ AUC at the limit of detection, which affects the theoretical reduction in AUC between vaccine and control groups. We did account for this by providing a semi-quantitative description of the reduction in shedding of RNA. Last, the results might have been biased towards positive findings; Out of the 13 vaccines that are on the market as of March 26, 2021 ^40^, we were only able to identify preclinical trials for eight in non-human primates. The absence of a complete overview could be an effect of a publication bias.

Our study implies that the lack of efficacy of many of the candidate vaccines to prevent infectiousness calls for caution, and one should be careful assuming that these vaccines prevent transmission. We interpret the limited reduction of viral RNA in the upper respiratory tract of some of the vaccine candidates as reason to consider individuals vaccinated with these vaccines to be potentially infectious after natural infection despite vaccination. However, interpreting the presence and reduction of viral RNA in the different samples as a measure of reduction of infectiousness depends on several assumptions that need to be validated or at least be made explicit. The evidence that comes from preclinical studies suffers from ‘indirectness’ ^41^. We do not measure the presence of infectious particles in vaccinated humans after natural infection, but measure viral RNA in artificially challenged NHPs. Despite that it is likely that viral RNA correlated with infectious virus, it remains unclear whether these RNA titres actually correspond with *de novo* produced infectious particles, or that a fraction of viral the RNA represents still input challenge virus ^38^. Some authors argued that subgenomic RNA might be more indicative of replication competent virus ^34, 42^, however, due to its stability, this might be an overestimate ^43^. We need to consider that the level of shedding might be age-dependent as well, since higher levels of SARS-CoV-2 RNA were detected in nasal swabs of older animals compared with younger animals ^10, 44^, thus the shedding might be more prominent in older individuals.

The results of the NHP challenge trials only allowed for a limited quantitative interpretation of the results in the context of the efficacy to prevent disease. The viral loads in lungs could be considered an indirect indicator of severity (lower viral loads may lead to milder lung pathology). In most studies, the vaccines did reduce either (histo)pathological changes or viral load in the lungs; the clinical changes, however, were often poorly assessed. Although NHP models might not always show severe COVID-19 disease ^10, 11^, Munster *et al.* presented a clinical score for NHP SARS-CoV-2 infection experiments ^11^, which showed that assessing clinical changes in this animal model is possible. From the assessed studies, only Doremalen *et al.* ^33^ quantified, using Munster *et al.* clinical score, the clinical effect of the challenge after vaccination. Liang *et al.*, despite referring to Muster *et al.*, only present the body weight as measure of clinical effectiveness ^25^. A meticulous assessment of clinical outcomes, as done for example by Doremalen *et al.*, does provide additional information for assessing the potential efficacy of vaccine candidates.

Our study also has several implications for the design and reporting of preclinical studies: First, it highlights the need for standardization. We found differences in almost all aspects of the preclinical trial design: In the timing, the dose, the strain and the administration route of the challenge; the diagnostic methods used, the selected timepoints at which the animals were sampled, and the assays used. These parameters influence the outcomes: For example, we showed here the relationship between the relative reduction in viral RNA shedding (efficacy) and the challenge dose and in the literature a more prominent viral replication in aged macaques than young macaques is described ^44^. Besides the properties of the experimental designs that we assessed, plenty other factors might influence the results: including serological- and/or microbiological status of the animals, the specifications of the challenge virus (RNA sequence heterogenicity and matching with the vaccine), sampling strategy, etc.. This all highlights a clear need for standardization and registration of protocols. Preregistration of the study and its protocol would allow others to harmonize and compare methods. Human trial registration has been the standard for over a decade ^45^. Second, it highlights the need to improve and standardize the quality of reporting: there is a heterogeneity in how the data is analysed and presented, adding another layer of difficulty to compare studies. Even within the same outcome, assay, and experiment, aggregating data and showing medians or means gives different results. The choice of the authors is often not transparent and seems ad hoc. Third, sharing research and data, preferably raw data, allows to make these comparisons more accurate. Especially during disease outbreaks, rapid open access sharing of manuscripts and its underlying data is of utmost importance ^46^. It does not only increase transparency and reproducibility, but helps tremendously in the interpretation and aggregation of data.

The development of SARS-CoV-2 vaccines is far from finished. New virus variants will continue to emerge and vaccines might become less effective against these new strains as was shown for the ChAdOx1 vaccine and its marked reduced efficacy against the B.1.351 variant ^47^. NHP trials might hold a key in predicting efficacy, however, only with standardized methods will studies be comparable and results could be used to be translated into guidance. Transmission experiments similar to those in ferrets ^48^, will help correlate persistence of viral RNA to actual infectiousness. Furthermore, we need to continue to improve our understanding on how the results of NHP models translate to ‘real world’ vaccine efficacy, in humans. For that, we need data. Importantly, as discussed above, we need to understand how effective these vaccines are in preventing infectiousness. We need carefully designed trials and observational studies ^5, 49^. We need to document the secondary attack rates originating from the vaccinated and unvaccinated groups; however, selective vaccination of risk groups complicates the interpretation of these comparisons. More advanced study designs, comparing populations with different vaccine coverages, could provide additional estimates of the vaccine efficacy on infectiousness ^49^. Early observations from Israel, where the vaccine roll-out has been rapid, reveals that individuals vaccinated with the BNT162b2 mRNA vaccine had markedly reduced RNA titres in the blood from 12 days post-vaccination. Consistent with these findings was an observed drop of the average CT values as vaccination roll-out progressed ^50^. These findings suggest a reduced infectiousness after vaccination.

## Conclusions

Preclinical challenge studies show that the majority of SARS-CoV-2 vaccines might not fully protect against infectiousness. Careful assessment of secondary attack rates from vaccinated individuals is warranted. Standardization of trial design and reporting will help contribute to the comparability and interpretation of preclinical trials.

## Data availability

### Underlying data

All data underlying the results are available as part of the article and no additional source data are required.

### Extended data

Open Science Framework: SARS-CoV-2 Vaccine efficacy Preclinical trials, https://doi.org/10.17605/OSF.IO/RDKMF. 

This project contains the following extended data:

- Data/DataSet_COVIDNHP.xlsx
- Data_Code.RprojOutput/Report_12april_rendered.html
- Scripts/COVRIN_Figures.R
- Scripts/main.R

### Reporting guidelines

Open Science Framework: PRISMA checklist and flow diagram,https://doi.org/10.17605/OSF.IO/RDKMF.

- PRISMA2009_checklist_sections.doc
- PRISMA_flowchart.docx

Data are available under the terms of the Creative Commons Zero "No rights reserved" data waiver (CC0 1.0 Public domain dedication).
