# Supplementary material for: Assessment of the efficacy of SARS-CoV-2 vaccines in non-human primate studies: a systematic review
Source: Open Res Eur. 2022 Jan 7;2:4. [Version 1] doi: 10.12688/openreseurope.14375.1 (PMC10446071; doi:10.12688/openreseurope.14375.1)
Supplement: Peer review history

# Peer review: Reviewer response for version 1

**DOI:** 10.21956/openreseurope.15514.r32576

**Published:** 2023-06-26

**Authors:** Eakachai Prompetchara

**Affiliations:** 1 Chulalongkorn University, Bangkok, Thailand

The authors did a very good job in this systematic review writing. The method of data collection, analysis, results analysis and presentation as well as conclusion are clearly demonstrated. 

 One of my suggestions is providing how authors derive each AUC cutoff for low (<18%), moderate (18–44%) and high (>44%) reduction of viral RNA excretion.

Are the rationale for, and objectives of, the Systematic Review clearly stated?

Yes

Is the statistical analysis and its interpretation appropriate?

Yes

Are sufficient details of the methods and analysis provided to allow replication by others?

Yes

Are the conclusions drawn adequately supported by the results presented in the review?

Yes

Reviewer Expertise:

Immunology, Virology, Animal model study

I confirm that I have read this submission and believe that I have an appropriate level of expertise to confirm that it is of an acceptable scientific standard.

# Peer review: Reviewer response for version 1

**DOI:** 10.21956/openreseurope.15514.r32585

**Published:** 2023-06-26

**Authors:** Georgia Kalodimou

**Affiliations:** 1 Division of Virology, Department of Veterinary Sciences, LMU Munich, Oberschleißheim, Germany; 2 Partner Site Munich, German Center for Infection Research, Oberschleißheim, Germany

The systematic review ‘Assessment of the efficacy of SARS-CoV-2 vaccines in non-human primate studies: a systematic review’ by Counotte *et al*. reviewed non-human primate (NHP) studies where animals were vaccinated with SARS-CoV-2 candidate vaccines and challenged with the virus. The authors focused on the ability of these vaccines to prevent transmission, by quantitatively analysing viral shedding after challenge in the chosen publications. Using this approach, they demonstrated that the vaccines showed significant differences in their ability to reduce viral RNA loads in different anatomical sites, with many inducing only low to moderate reductions.

 The study also highlighted the heterogeneity of SARS-CoV-2 vaccine/challenge studies in NHP in terms of the study protocol, the reporting of viral RNA measurements and the reporting of clinical and pathological outcomes. The authors discussed the difficulty of comparing the efficacy of different vaccines when factors such as clinical and pathological outcomes are not reported thoroughly. Thus, the manuscript underlines the importance of standardising the design and reporting of pre-clinical NHP studies. It also raises important questions on the ability of many SARS-CoV-2 vaccines to protect against infectiousness.

 Overall, the manuscript is well written, has a good structure and is easy to understand. The authors clearly state the importance of performing the analysis and highlight the challenge of comparing studies that are not standardised. The methodology used by the authors is appropriate for this type of study in regard to selection, data extraction and analysis. The tables in the main body of the manuscript and the supplement used to describe the features of the chosen studies are clear, detailed and understandable. While the figures enable the reader to conceptualise the differences between the NHP studies analysed.

 I believe that the manuscript is suitable for indexing and does not require major revisions. There are however a few minor issues that need to be addressed, which are listed below.

- The authors refer to Supplementary Material Table X and Supplementary Material Figure X but in the supplementary section, they are titles Supplementary Table X and Supplementary Figure X. Please remove the word ‘Material’ when referring to the supplementary tables and figures in the manuscript.
- Results section ‘Description of the included studies’ paragraph 1, line 4 (page 5): Change ‘147’ to ‘141’, the number in the PRISMA Flow Chart.
- Results section ‘Description of the included studies’ paragraph 2, line 2 (page 5): Change ‘experiment’ to ‘experiments’.
- Results section ‘Description of the included studies’ paragraph 2, line 4 (page 5): Please add a full stop after ‘macaques’.
- Discussion paragraph 3, line 15 (page 8): Change ‘halve’ to ‘half’.
- Supplementary Figures 1, 3 and 4 are missing figure legends. Please add them.

Are the rationale for, and objectives of, the Systematic Review clearly stated?

Yes

Is the statistical analysis and its interpretation appropriate?

Yes

Are sufficient details of the methods and analysis provided to allow replication by others?

Yes

Are the conclusions drawn adequately supported by the results presented in the review?

Yes

Reviewer Expertise:

virology, vaccine immunology, infection immunity

I confirm that I have read this submission and believe that I have an appropriate level of expertise to confirm that it is of an acceptable scientific standard.
